# Mother and Child T Cell Receptor Repertoires: Deep Profiling Study

**DOI:** 10.3389/fimmu.2013.00463

**Published:** 2013-12-25

**Authors:** Ekaterina V. Putintseva, Olga V. Britanova, Dmitriy B. Staroverov, Ekaterina M. Merzlyak, Maria A. Turchaninova, Mikhail Shugay, Dmitriy A. Bolotin, Mikhail V. Pogorelyy, Ilgar Z. Mamedov, Vlasta Bobrynina, Mikhail Maschan, Yuri B. Lebedev, Dmitriy M. Chudakov

**Affiliations:** ^1^Shemyakin-Ovchinnikov Institute of Bioorganic Chemistry, Russian Academy of Science, Moscow, Russia; ^2^Federal Scientific Clinical Center of Pediatric Hematology, Oncology and Immunology, Moscow, Russia; ^3^Central European Institute of Technology (CEITEC), Masaryk University, Brno, Czech Republic

**Keywords:** TCR repertoires, NGS, maternal-fetal exchange, public clonotypes, T cell receptor, haploidentical transplantation, autoimmune diseases, microchimerism

## Abstract

The relationship between maternal and child immunity has been actively studied in the context of complications during pregnancy, autoimmune diseases, and haploidentical transplantation of hematopoietic stem cells and solid organs. Here, we have for the first time used high-throughput Illumina HiSeq sequencing to perform deep quantitative profiling of T cell receptor (TCR) repertoires for peripheral blood samples of three mothers and their six children. Advanced technology allowed accurate identification of 5 × 10^5^ to 2 × 10^6^ TCR beta clonotypes per individual. We performed comparative analysis of these TCR repertoires with the aim of revealing characteristic features that distinguish related mother-child pairs, such as relative TCR beta variable segment usage frequency and relative overlap of TCR beta complementarity-determining region 3 (CDR3) repertoires. We show that thymic selection essentially and similarly shapes the initial output of the TCR recombination machinery in both related and unrelated pairs, with minor effect from inherited differences. The achieved depth of TCR profiling also allowed us to test the hypothesis that mature T cells transferred across the placenta during pregnancy can expand and persist as functional microchimeric clones in their new host, using characteristic TCR beta CDR3 variants as clonal identifiers.

## Introduction

Closeness and relationship between mother and child immunity have been the focus of studies of pregnancy (1), autoimmunity (2–4) and haploidentical transplantations of hematopoietic stem cells (HSCs) (5), and solid organs (6, 7).

In recent years, the potential of next-generation sequencing (NGS) to reveal the full complexity of human and mouse immune receptor repertoires has inspired numerous efforts to develop optimal techniques for achieving large-scale T cell receptor (TCR) and antibody profiling (8–12) and to decipher various aspects of adaptive immunity (8, 9, 11, 13–17). With appropriate library preparation methods (18), NGS techniques now make it possible to perform quantitative analysis of hundreds of thousands or millions of distinct TCR beta complementarity-determining region 3 (CDR3) variants. This individual diversity of TCR beta CDR3 variants, which is generated in the course of V-D-J recombination and the random addition and deletion of nucleotides in the thymus, largely determines the whole diversity of naïve T cells and specificity of T cell immune responses (19, 20).

In the present study, we have used deep NGS profiling to compare TCR beta repertoires of mothers and their children. We achieved a profiling depth of 500,000–2,000,000 unique TCR beta CDR3 clonotypes per donor, and performed comparative analysis with the aim of revealing specific features of TCR repertoires that distinguish related mother-child pairs from unrelated individuals, and how these familial repertoires manifest the influence of inherited factors, such as the elements of TCR recombination machinery and human leukocyte antigens (HLA). By comparing out-of-frame (i.e., non-functional and thus not subjected to selection) and in-frame TCR beta repertoires, we also show the extent of the impact of thymic selection and the common trends in how this process shapes individual repertoires.

Additionally, the profiling depth that we achieved allowed us to look for the potential presence of maternal or fetal microchimeric T cell clones that may have transmigrated through the placenta as mature α/β T cells and which subsequently persist in both related donors, by using characteristic TCR beta CDR3 variants as clonal identifiers.

## Materials and Methods

### Sample collection

This study was approved by the ethical committee of the Federal Scientific Clinical Center of Pediatric Hematology, Oncology, and Immunology. Blood donors provided informed consent prior to participating in the study. Ten milliliters of peripheral blood samples were obtained from nine systemically healthy Caucasian donors: three mothers (average age 40 ± 4 years) and their six children (average age 11 ± 4 years). Peripheral blood mononuclear cells (PBMCs) were isolated by Ficoll-Paque (Paneco, Russia) density gradient centrifugation. Total RNA was isolated with Trizol (Invitrogen, USA) in accordance with the manufacturer’s protocol.

### Contamination precautions

T cell receptor beta libraries were generated in clean PCR hoods with laminar flow, using reagents of high purity and pipette tips with hydrophobic filters. As an additional precaution, we generated the TCR beta libraries for the two groups being compared – mothers and their children – a month apart, and sequenced the two libraries in two separate Illumina runs to guarantee the absence of inter-library contamination during amplification or on the solid phase of the sequencer.

### Preparing cDNA libraries for quantitative TCR beta profiling

cDNA-based library preparation was performed essentially as described previously (9, 12, 16, 18, 21, 22). Briefly, we used the Mint kit (Evrogen, Russia) for first-strand cDNA synthesis. For each donor sample, the whole amount of extracted RNA was used for cDNA synthesis, with 1.5 μg of RNA per 15 μl reaction volume. We incubated the mixture of RNA and priming oligonucleotide BC_R4_short (GTATCTGGAGTCATTGA), which is specific to both variants of the human TCR beta constant (TRBC) segment, at 70°C for 2 min and 42°C for 2 min for annealing. We then added the 5′-adapter for the template switch. The reaction was carried out at 42°C for 2 h, with 5 μl of IP solution added after the first 40 min.

Further cDNA library amplification was performed in two sequential PCRs using Encyclo PCR mix (Evrogen). To capture the maximum number of input cDNA molecules, we used the whole amount of synthesized cDNA for the first PCR amplification. The first PCR totaled 18 cycles with universal primers M1SS (AAGCAGTGGTATCAACGCA) and BC2R (TGCTTCTGATGGCTCAAACAC), which are respectively specific to the 5′-adapter and a nested region of the TRBC segments. The primer annealing temperature was set at 62°C. The products of the first PCR were combined, and a 100-μl aliquot was purified by QIAquick PCR purification Kit (Qiagen) and eluted by 20 μl of EB buffer.

The second PCR amplification was performed for 8–10 cycles with a mix of TCR beta joining (TRBJ)-specific primers and the universal primer M1S ((N)_2–4_(XXXXX)CAGTGGTATCAACGCA GAG), which is specific to the 5′-adapter and is nested relative to the M1SS primer used in the first PCR amplification. XXXXX represents a sample barcode introduced in the second PCR, and (N)_2–4_ are random nucleotides that were added in order to generate diversity for better cluster identification during Illumina sequencing. Primer annealing temperature was set at 62°C.

### Illumina HiSeq sequencing

PCR products carrying pre-introduced sample barcodes were mixed together in equal ratio for each of the two groups (mothers and children). Illumina adapters were ligated according to the manufacturer’s protocol using NEBNext DNA Library Prep Master Mix Set for Illumina (New England Biolabs, USA). Generated libraries were analyzed using two separate Illumina HiSeq 2000 lanes in separate runs with 100 + 100 nt paired end sequencing using Illumina sequencing primers. Raw sequences deposited in NCBI SRA database (PRJNA229070).

### NGS data analysis

TCR beta variable (TRBV) segment identification [using IMGT nomenclature (23)], CDR3 identification (based on the sequence between conserved Cys-104 and Phe-118, inclusive), clonotype clusterization and correction of reverse transcription, PCR, and sequencing errors were performed using our MiTCR software (24)^1^. The sequencing quality threshold of each nucleotide within the CDR3 region was set as Phred >25, with low-quality sequence rescue by mapping to high-quality clonotypes. The strictest “eliminate these errors” correction algorithm was employed to eliminate the maximal number of accumulated PCR and sequencing errors.

### Statistical analysis

We used Jensen–Shannon divergence (JS), which is a symmetrized version of the Kullback–Leibler divergence (KL), to quantify the similarity between the clonotype TRBV gene usage distribution in related and unrelated mother-child pairs. JS and KL are defined as follows (25):

$$\begin{array}{llll} \mathrm{JS}\left(P,Q\right) & =\frac{1}{2}\left(\mathrm{KL}\left(P,\;\frac{P+Q}{2}\right)+\mathrm{KL}\left(Q,\;\frac{P+Q}{2}\right)\right),\; & & \;\\ \mathrm{KL}\left(P,Q\right) & =\sum_{i}p_{i}\log_{2}\frac{pi}{qi}.\; & & \; \end{array}$$

Where *P* and *Q* correspond to the TRBV gene segment frequency distributions of the two individuals being analyzed, and *p_i_* and *q_i_* stand for the frequency of a particular TRBV gene segment in the first and second individual, correspondingly. For statistical comparison of the JS among related and unrelated mother-child pairs, we used two-tailed, unpaired Student’s *t*-test with *P*-values <0.05 considered significant. To account for multiple testing, Bonferroni-corrected *P*-values were used.

We used linear regression to analyze dependency between TRBV-CDR3/CDR3 overlap ratio and the number of shared major histocompatibility complex I (MHC-I) alleles, and calculated the Pearson correlation coefficient. The linear model:

$$\begin{array}{llll} & \mathrm{TRBV -CDR3∕CDR3}\;\mathrm{overlap}\;\mathrm{ratio}=b0+b1\; & & \;\\ & \;\times \left[\mathrm{Number}\;\mathrm{of}\;\mathrm{shared}\;\mathrm{MHC}\;\mathrm{alleles}\right]\; & & \; \end{array}$$

was fit using the least-squares method. Linear regression and correlation analysis were performed using *R* programing language^2^.

### FACS analysis

We used the following anti-human antibodies: CD3-PC7 (clone UCHT1, eBioscience), CD27-PC5 (clone 1A4CD27, Invitrogen), CD4-PE (clone 13B8.2, Beckman Coulter), CD45RA FITC (eBioscience, clone JS-B3). An aliquot of PBMC was incubated with antibodies for 20 min at room temperature, washed twice with PBS and analyzed via Cytomics FC 500 (Beckman Coulter).

### HLA typing

The samples were HLA-typed using SSP AllSet Gold HLA-ABC Low Res Kit and SSP AllSet Gold HLA-DRDQ Low Res Kit (Invitrogen) and results were processed using UniMatch software.

## Results

We obtained at least 1 × 10^7^ TCR beta CDR3-containing sequencing reads for each mother and about 3 × 10^6^ reads for each child. MiTCR software analysis yielded 500,000–2,000,000 distinct TCR beta CDR3 clonotypes per donor (Table 1) – representing a significant portion of the total TCR beta diversity for an individual, which lower bound estimate constitutes ~4 million (8). We then subjected these individual TCR beta datasets to comparative analysis in an effort to identify features that distinguish TCR beta repertoires of related mother-child pairs.

**Table 1:** **Sequencing results: TCR beta reads and clonotypes**.

Donor	Sex	Age	Paired end sequencing reads	TCR beta CDR3- containing reads (%)	TCR beta clonotypes before error correction	Final TCR beta clonotypes	% Of out-of-frame clonotypes of all clonotypes	% Of naïve T cells of all T cells (FACS analysis)	% Occupied by >0.001% clonotypes, of all reads
Mother A	F	36	13,656,054	12,513,543 (91.6)	2,044,290	1,708,037	2.7	55.0	19.4
Mother B	F	43	13,872,805	12,901,795 (93.0)	1,213,738	918,557	2.6	27.3	46.2
Mother C	F	43	11,167,059	10,038,463 (89.9)	2,180,886	1,978,745	3.0	39.4	38.2
Child A1	M	11	4,687,578	2,889,352 (61.6)	756,772	729,800	2.6	57.2	16.4
Child A2	M	9	4,202,419	2,467,388 (58.7)	558,173	535,283	2.4	43.0	28.2
Child B1	M	16	4,830,536	3,173,376 (65.7)	821,908	790,592	3.2	60.9	17.2
Child B2	M	10	4,615,093	3,009,961 (65.2)	545,730	517,410	2.3	40.1	34.3
Child C1	F	6	6,081,365	3,940,367 (64.8)	1,104,982	1,060,854	2.3	73.7	13.9
Child C2	M	16	4,564,646	3,008,748 (65.9)	785,164	760,572	2.6	63.7	19.7

### TRBV gene usage

**Figure 1:**
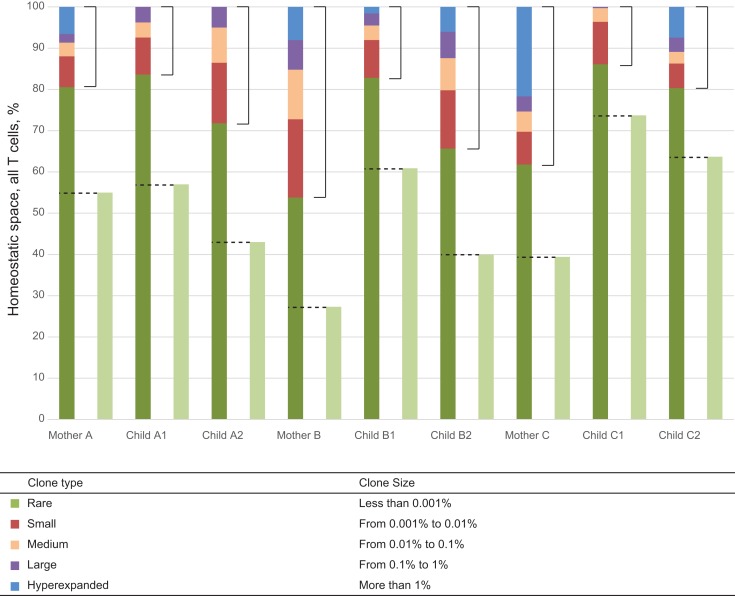
**Representation of T cell clones of different size in individual TCR beta repertoires**. Colored bars represent the share of clonal space occupied by clones of given type (classified by size) for each of the nine donors. Light green bars represent the share of naïve CD27^high^CD45RA^high^ T cells as determined by FACS analysis. Dashed lines indicate the share of low-frequency TCR beta clonotypes equivalent to this population in each individual, which were included in “low-frequency in-frame clonotypes” analysis. Square brackets indicate the share occupied by high-frequency T cell clones each representing >0.001% of all T cells.

We analyzed the relative usage of TRBV gene segments in mother-child pairs at three levels (see Figure 1):

#### Out-of-frame TCR beta variants

The influence of genetic effects on the recombination machinery, which determines the relative frequencies of TRBV gene segment usage in TCRs generated before selection in the thymus, should be reflected by out-of-frame TCR variants that are not subjected to the pressure of further selective processes. Due to nonsense-mediated decay mechanisms, RNA-based libraries generally contain a low percentage of out-of-frame TCR beta variants (9, 12, 26, 27). Nevertheless, out-of-frame CDR3 sequences constituted ~2.5% of all clonotypes (Table 1) – 16,048–45,300 clonotypes per donor – which is sufficiently abundant to perform statistical analysis. These subsets were used to compare TRBV gene segment usage in related and unrelated mother-child pairs before thymic selection.

**Figure 2:**
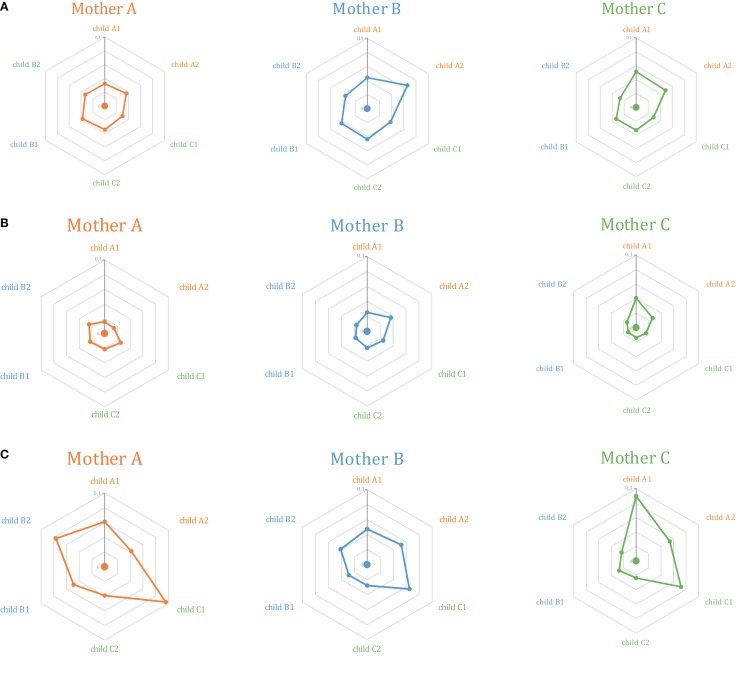
**Relative similarity of TRBV gene segments usage analyzed using the Jensen–Shannon divergence method for (A) out-of-frame TCR beta variants; (B) Low-frequency in-frame TCR beta clonotypes; and (C) high-frequency in-frame TCR beta clonotypes**. The central dot in each diagram represents Mother **(A)** (orange), **(B)** (blue), or **(C)** (green). Surrounding dots represent the six children. Related children are shown in the same color as their mothers. The closer the “child” dot is to the central “mother” dot, the lower the Jensen–Shannon divergence (i.e., more similar TRBV gene segment usage).

**Figure 3:**
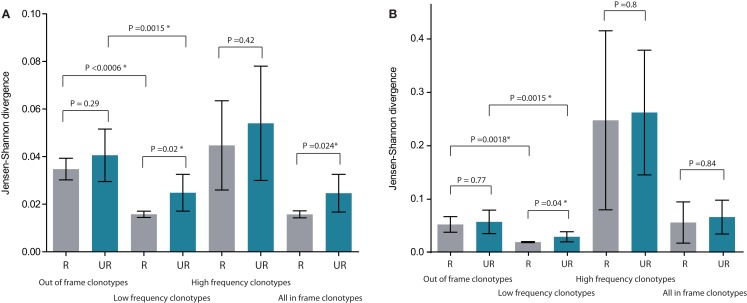
**Mean Jensen–Shannon divergence for TRBV gene segment usage for related (R, gray) versus unrelated (UR, blue) mother-child pairs, with SD**. Bonferroni-corrected *P*-values are provided to account for multiple testing. **(A)** Comparison of TRBV gene segment usage frequencies at the level of distinct TCR beta clonotypes (but not sequencing reads, so that the influence of relative TCR beta clonotype size within a given repertoire is excluded). **(B)** Comparison of TRBV gene segment usage frequencies at the level of sequencing reads, i.e., considering relative abundance of clonotypes in peripheral blood. The lower the Jensen–Shannon divergence, the more similar the TRBV gene segment usage. *Denotes statistical significance. See Figure 1 for delineation of low- and high-frequency clonotypes. Please note that “All in-frame clonotypes” include not only low-frequency and high-frequency clonotypes, but also the medium-frequency ones.

At this level of out-of-frame non-functional TCR beta variants, Jensen–Shannon divergence in TRBV gene usage was comparable for related and unrelated mother-child pairs, albeit with a non-significant increase in divergence for the latter (Figure 2A; Figures 3A,B, first 2 bars).

#### Low-frequency in-frame clonotypes

The pressure of thymic selection can be tracked by comparing TRBV gene segment usage in out-of-frame TCR beta variants relative to those variants represented in naïve T cells. In this work, we did not perform separate TCR profiling of FACS-sorted naïve T cells. We aimed to achieve maximal depth of analysis, and sought to avoid the loss of cells and RNA and general quantitative biases that inevitably arise from the cell sorting process. We estimated the pool of TCR beta clonotypes that predominantly belong to the naïve subset as follows. We used FACS analysis to identify the percentage of naïve CD27^high^CD45RA^high^ CD3+ T cells for each donor (28). This analysis demonstrated that naïve T cells constitute 40–73% of the T cell population in children and 27–55% of the T cell population in mothers (Table 1; Figure 1). Since each naïve T cell clone is usually represented by minor numbers of TCR-identical cells in an individual (29), for the purposes of bulk analysis, we hypothesized that the subset of the low-frequency clonotypes that occupies the same share of homeostatic space as the FACS-determined share of naïve T cells for that particular donor (433,293–1,797,650 clonotypes per donor) predominantly includes naïve T cells.

**Figure 4:**
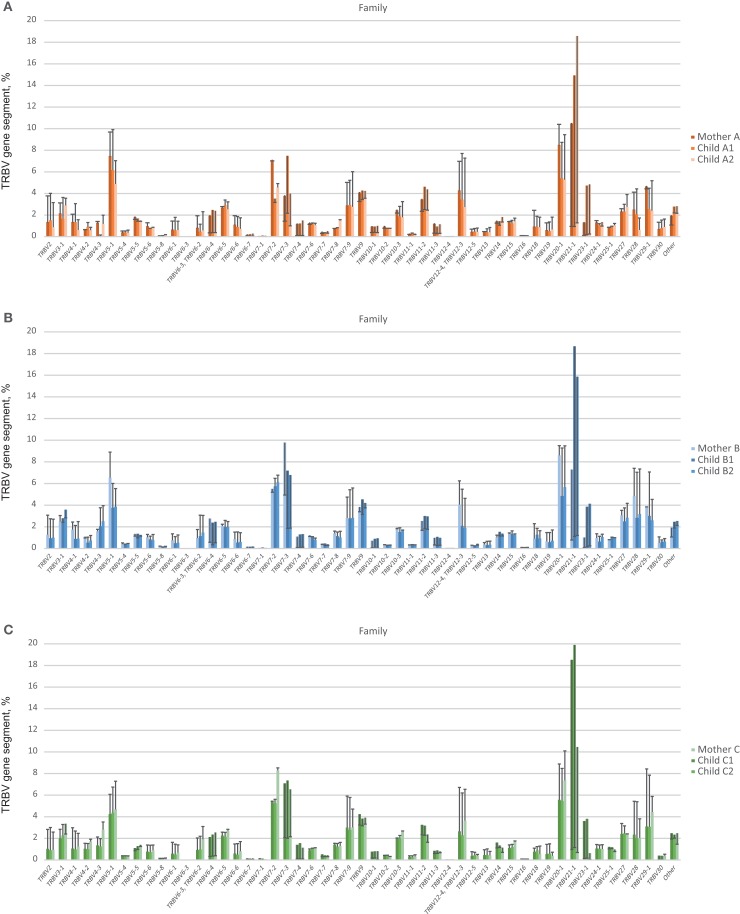
**TRBV gene segment usage in functional low-frequency TCR beta clonotypes in comparison to out-of-frame TCR variants**. Colored bars indicate the representation of a particular TRBV gene segment family in out-of-frame TCR variants from each individual. Lines represent alterations in TRBV gene segment representation in functional low-frequency TCR beta clonotypes relative to out-of-frame TCR variants. **(A)**, **(B)**, and **(C)** depict TRBV gene segment usage for related donors from family **(A)**, **(B)**, and **(C)**, respectively.

At this level of low-frequency, in-frame TCR beta clonotypes, TRBV gene segment usage was significantly less divergent compared to out-of-frame TCR beta variants, both in related and unrelated pairs (Figures 2B and 3). Additionally, TRBV gene segment usage was significantly more similar for related versus unrelated pairs (Figures 3A,B, bars 3, 4). In accordance with JS analysis, comparison within related triplets revealed equalization of the usage of particular TRBV gene segments in low-frequency, in-frame TCR clonotypes compared to out-of-frame TCR variants (Figure 4). For example, in each triplet, we saw the usage of TRBV gene segments 12-3, 12-4, 20-1, 21-1, and 23-1 equalize in the low-frequency TCR beta clonotypes pool. We also observed an equalizing decrease in TRBV 7-3 usage in triplets A and C, and an equalizing increase in TRBV 28 usage in triplet B.

Notably, the observed changes in TRBV gene segments usage were generally similar in different unrelated donors (compare Figures 4A–C), and the convergence of TRBV usage after thymic selection (difference of out-of-frame versus in-frame TRBV usage divergence) was not significantly dependent on the number of shared HLA alleles (*R* = 0.12, *P* = 0.63).

#### High-frequency in-frame clonotypes

The influence of antigen-specific reactions on selection of TRBV gene segments could be tracked by comparing TRBV gene usage in naïve and antigen-experienced T cells. Following the same logic that we used above for the approximate identification of the subset of naïve TCR beta clonotypes, we hypothesized that the most abundant clonotypes predominantly represent antigen-experienced T cell clones. We defined this population as clones representing >0.001% of all CDR3 sequences. Thus, the lower bound for this group was approximately an order of magnitude greater than the upper border set for the low-frequency clones in a given donor’s T cell pool (Figure 1). Such delineation with a gap between the two subsets minimized “contamination” by naïve TCR beta clonotypes. Still, the pool of high-frequency in-frame clonotypes could contain a portion of naïve clonotypes with TCR beta CDR3 sequence variants of low complexity, that are repetitively produced in thymus due to the convergent recombination events and thus may be highly represented (15).

This set of the 2,803–8,285 most abundant clonotypes per individual cumulatively occupied 13.9–46.2% of the homeostatic T cell space in each donor. These high-frequency TCR beta clonotypes were generally characterized by increased variability in TRBV gene segment usage, and related and unrelated mother-child pairs were nearly indistinguishable (Figure 2C; Figures 3A,B, bars 5, 6).

### Overlap of TCR beta repertoires for related and unrelated mother-child pairs

Several studies in recent years have revealed that unrelated individuals widely share TCR beta repertoires (13–15, 30–32). However, it is presently unclear whether the repertoires of haploidentical individuals are characterized by a higher level of overlap compared to unrelated donors. Additionally, for related mother-child pairs, shared TCR beta variants could conceal microchimeric T cell clones that have been physically shared across the placenta (see below).

To address these questions, we performed comparative analysis of TCR beta repertoire overlap for related and unrelated mother-child pairs by quantifying CDR3 variant identity at the amino acid level, at the nucleotide level, and at the nucleotide level in conjunction with identical TRBV and TRBJ gene segment usage (i.e., fully identical TCR beta chains). We measured overlaps separately for low-frequency and high-frequency in-frame clonotypes (as delineated in Figure 1), and all in-frame clonotypes. Table 2 shows raw, non-normalized numbers of CDR3 variants shared on average by related and unrelated mother-child pairs.

**Table 2:** **Average number of shared TCR beta CDR3 clonotypes in related and unrelated pairs**.

Pairs	Amino acid			Nucleotide			Nucleotide TRBV, TRBJ identical		
	Low- frequency	High- frequency	All clonotypes	Low- frequency	High- frequency	All clonotypes	Low- frequency	High- frequency	All clonotypes
Related	71,714 ± 25,890	117 ± 34	83,257 ± 26,056	4,938 ± 2,237	69 ± 7	8,407 ± 3,396	2,015 ± 893	40 ± 10	3,456 ± 1,359
Unrelated	68,979 ± 18,822	99 ± 15	80,304 ± 18,626	4,640 ± 1,587	59 ± 12	7,955 ± 2,227	1,823 ± 624	31 ± 11	3,135 ± 897

For comparative analysis of relative overlap between subsets of different size, we normalized the number of identical CDR3 variants based on the sizes of the cross-compared samples as follows:

$$\begin{array}{l} \text{Formula }1:\;[\text{normalized overlap between TCR sets }A\text{ and }B]\\ \;=\;[\text{overlap between TCR sets }A\text{ and }B\text{]/}([\text{number of}\\ \text{ clonotypes in set }A]\;\times \;[\text{number of clonotypes in set }B]). \end{array}$$

**Figure 5:**
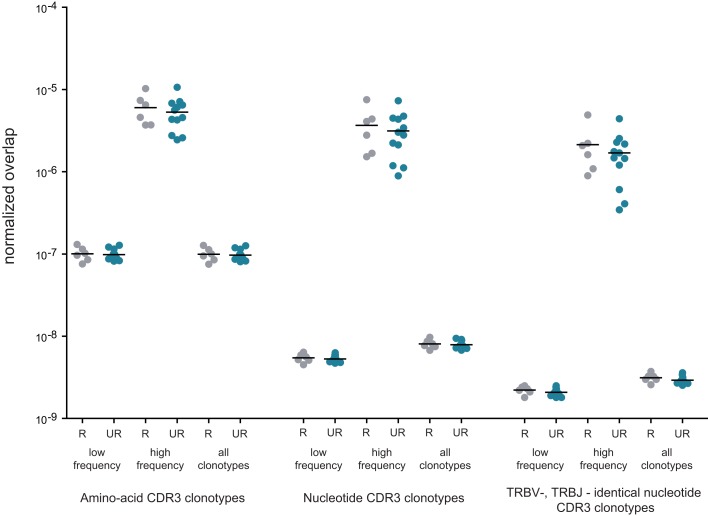
**Normalized overlap of individual TCR beta repertoires**. Overlaps are shown at the level of CDR3 amino acid sequences, nucleotide sequences, and nucleotide sequences with identical TRBV and TRBJ segments; for related (gray) versus unrelated (blue) mother-child pairs; and for low-frequency, high-frequency, and all in-frame clonotypes. The number of intersections was normalized as described in Formula 1. R, related pairs; UR, unrelated pairs.

Normalized results are plotted in Figure 5. For all CDR3 categories, the degree of overlap was always slightly higher for related pairs, but this difference never approached a significant level compared to unrelated pairs. The highest level of overlap was observed for high-frequency clonotypes, in agreement with the previous work (15).

### Within amino acid CDR3 overlaps of expanded clonotypes, percentage of clonotypes with identical TRBV genes is increased for related mother-child pairs

The CDR3 region is considered to form interactions mainly with antigenic peptide, while CDR1 and CDR2 encoded in the TRBV segment are mostly responsible for MHC recognition (33–35). Some TRBV segments have nearly identical sequences taking part in CDR3 formation, so two different TRBV segments can often give rise to the same CDR3 amino acid sequence. However, in two individuals with similar or identical HLA alleles, proliferating antigen-specific clones with the same TRBV segment and CDR3 amino acid sequence that recognize the same peptide-MHC complex can be preferentially activated (36). Therefore, since related mother and child pairs share at least 50% of their HLA alleles, we could expect that antigen-experienced clones with identical amino acid CDR3 variants that recognize the same antigenic peptide should more often carry the same TRBV segment encoding CDR1 and CDR2 responsible for MHC recognition.

**Figure 6:**
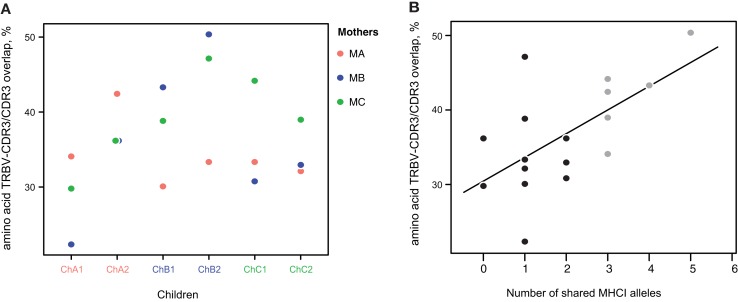
**Amino acid TRBV-CDR3/CDR3 overlap ratio**. **(A)** The ratio of TRBV-CDR3 overlap to CDR3 overlap for all possible mother-child pairs, based on the 10,000 most highly represented clonotypes from each donor. Related mother-child pairs had a higher ratio relative to children with either of the unrelated mothers. **(B)** The number of shared MHC-I alleles in mother-child pairs correlates with the TRBV-CDR3/CDR3 overlap ratio for the 10,000 most abundant CDR3 clonotypes. Solid line displays linear regression fit; the Pearson correlation coefficient was 0.62 (*P* < 0.006). Related and unrelated pairs are shown in gray and black, respectively.

To verify this hypothesis, we analyzed various repertoire pairs comprising the 10,000 most abundant amino acid CDR3 clonotypes from each individual and computed overlap in terms of shared amino acid CDR3 sequences and shared amino acid CDR3 sequences carrying the same TRBV segment (i.e., identical CDR1, 2, and 3). We then determined the ratio of TRBV-CDR3 overlap to CDR3 overlap for each mother-child pair. In all cases, the ratio was greater for related mother-child pairs (1.3-fold, ±0.16, Figure 6A). Moreover, we observed significant positive correlation of this ratio with the number of shared MHC-I alleles between individuals (*R* = 0.62, *P* < 0.006, Figure 6B; Table 3).

**Table 3:** **HLA typing**.

	HLA-A	HLA-B	HLA-C	DRB1	DRB3	DRB4	DQB1
Mother A	02, 24	15, 57	03, 06	07, 14	01–03	01	03, 05
Mother B	02, 23	44, 51	02, 04	07, 11	01–03	01-02	02, 03
Mother C	01, 11	08, 35	04/08, 07	01, 03	01–03	–	02, 05
Child A1	02, 25	15, 18	03, 12	04, 14	01–03	01–02	03, 05
Child A2	02, 02	15, 44	03, 05	11, 14	01–03	–	03, 05
Child B1	02, 23	38, 44	04, 12	07, 13	01–03	01–02	02, 06
Child B2	02, 23	27, 44	02, 04	07, 11	01–03	01–02	02, 03
Child C1	02, 11	35, 38	04, 12	01, 13	01–03	–	05, 06
Child C2	02, 11	35, 38	04, 12	01, 13	01–03	-	05, 06

### Selection in the thymus decreases average CDR3 length compared to the initially generated repertoire

**Figure 7:**
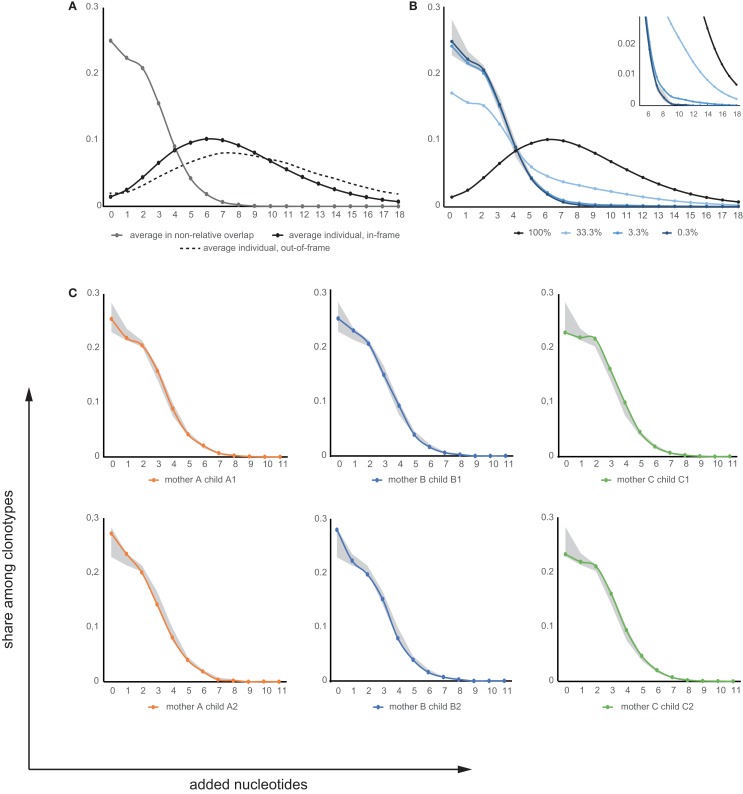
**Added nucleotide curves**. **(A)** Average distribution of added nucleotides within CDR3 for individual TCR beta repertoires (in-frame: black solid line; out-of-frame: black dashed line) and for TCR beta clonotypes shared between unrelated individuals (gray). **(B)** Modeling of added nucleotide curves for shared TRBV-CDR3-TRBJ variants between mother-child pairs based on input of mature-microchimeric TCR beta CDR3 variants in different proportions. This was derived from the curves in **(A)**, which depict added nucleotide distributions for shared clonotypes between unrelated individuals (gray; equivalent to near-zero contribution to shared clonotypes) and for any individual repertoire (black; equivalent to 100% contribution to shared clonotypes), mixed in different proportions. Lines represent model input where mature-microchimeric TCR beta is equal to 100, 33, 3.3, or 0.3% of shared clonotypes. Shaded region shows the range for unrelated pairs. Inset shows a magnified view. **(C)** Added nucleotide curves for TRBV-CDR3-TRBJ variants shared in each related mother-child pair. Shaded region shows the range for unrelated pairs.

Comparison of the out-of-frame and in-frame CDR3 repertoires revealed that the former are characterized by higher average length (45.6 ± 0.4 versus 43.3 ± 0.2) and an increased number of added nucleotides (8.6 ± 0.2 versus 7.4 ± 0.1, see Figure 7A), in both mothers and children.

This finding indicates that, upon recombination, the initially generated TCR beta CDR3 repertoire (the parameters of which are preserved in the non-functional out-of-frame repertoire) is characterized by higher average length, while further selection in thymus essentially shapes the repertoire toward lower CDR3 length and fewer added nucleotides.

### Searching for microchimeric clones transferred across the placenta as mature T cells

It is well established that mother and child exchange cells across the placenta during pregnancy (37–42), and that the progeny of these migrating cells persist in the new host for decades after gestation (43–45).

Most authors agree that lymphoid progenitor cells commonly cross the placenta to populate the new host (45–48). Some observations also indicate that mature T cells can transmigrate through the placenta (see Discussion). However, it remains to be determined whether the transferred mature T cells (hereinafter referred to as mature-microchimeric T cells) can further persist and serve as functional T cell clones in their new host.

We hypothesized that the present deep sequence analysis of such a substantial portion of the maternal and fetal TCR repertoire (including the absolute majority of proliferated antigen-experienced T cell clones) could reveal the presence of transferred and multiplied functional T cell populations, albeit without the immediate ability to distinguish the direction of transfer (i.e., maternal versus fetal microchimerism). Indeed, microchimeric T cell clones that were initially transferred across the placenta as mature T cells (mature-microchimeric T cell clones) within a given mother-child pair should be characterized by the same TCR beta CDR3 nucleotide sequence and the same TRBV and TRBJ gene segments, which therefore could serve as a clone-specific identifier.

However, ~40% of the CDR3 nucleotide variants shared between any two individuals were characterized with the same TRBV and TRBJ gene segments, in similar numbers for both related and unrelated mother-child pairs. This means 1,766–5,410 shared clonotype variants across different donor pairs (Table 2; Figure 5). This widespread sharing of identical TCR beta nucleotide variants makes the TRBV-CDR3-TRBJ identifier insufficient to distinguish clones that were physically transferred across the placenta as mature T cells with recombined TCRs from public TCRs resulting from independent convergent recombination events (15, 32). Thus, if mature-microchimeric T cell clones are present, they are concealed amongst the overwhelming majority of natural public TCRs, and additional characteristics are needed to delineate them.

It has been reported that public TCR beta clonotypes are generally characterized by a low number of added nucleotides in CDR3 (i.e., low complexity) (14, 15, 32). We therefore used the number of added nucleotides as an additional selective characteristic that essentially determines the probability of convergent recombination events leading to CDR3 variants that are identical at the nucleotide level (32, 49). Comparison of this characteristic for all TCR beta CDR3 nucleotide variants and those TRBV-CDR3-TRBJ nucleotide variants that were shared between unrelated mother-child pairs revealed that the latter were characterized by much lower numbers of added nucleotides (Figure 7A).

The transfer of mature T cells across the placenta should not be dependent on CDR3 length or the number of added nucleotides. In humans, it has been demonstrated that there is no significant difference between adult blood and cord blood samples in the mean number of added nucleotides (50). Therefore, this characteristic should be essentially identical for both feto-maternal and materno-fetal mature-microchimeric T cell clones and for the general TCR beta repertoire. If the TCR beta repertoires of related mother-child pairs carry mature-microchimeric T cell clones of interest, we would expect to observe shaping of the added nucleotide curve proportional to the contribution of such clones to the repertoire overlap (Figure 7B).

The sensitivity of this method to the percentage of mature-microchimeric T cell clones in the shared TCR beta population is therefore limited by the natural dispersion of the added nucleotide curves for unrelated pairs. For example, if mature-microchimeric T cell clones contribute ~0.3% of the TRBV-CDR3-TRBJ overlap for a mother-child pair (i.e., ~10 out of 3,000 overlapping clonotypes, out of the ~1 × 10^6^ total clonotypes sequenced from each donor), the shape of the added nucleotide curve would be indistinguishable from that of an unrelated donor pair – and therefore below the sensitivity threshold of this method. In contrast, the presence of 100 mature-microchimeric T cell clones out of 3,000 clonotypes (i.e., 3.3% of shared variants) per pair of related donors could be clearly distinguished (Figure 7B), and this can therefore be considered as the approximate sensitivity limit of the method. We subsequently determined that the presence of mature-microchimeric T cell clones is undetectable in all cases, based on the added nucleotide curves for overlapping TRBV-CDR3-TRBJ nucleotide sequences for our six related mother-child pairs (Figure 7C). Correspondingly, the average numbers of added nucleotides in the shared TRBV-CDR3-TRBJ nucleotide variants were indistinguishable for related versus unrelated mother-child pairs (data not shown).

The above-described comparison of added nucleotide curves was performed at the level of distinct TCR beta clonotypes, but not sequencing reads, so that the influence of each T cell clone’s relative representation within the repertoire was excluded. Similar albeit noisier results we have obtained when performing the same analysis at the level of sequencing reads (i.e., taking into account relative clonal size).

As such, we have not identified any meaningful difference between the subsets of shared TRBV-CDR3-TRBJ nucleotide variants for related versus unrelated mother-child pairs that would allow us to establish detection of a subpopulation of mature-microchimeric T cells that have been systemically shared during pregnancy as mature naïve or memory T cells, and which subsequently have engrafted and survived for years.

## Discussion

### TRBV gene usage

For out-of-frame TCR beta variants, which are not expressed and thus avoid any selection, TRBV gene usage was slightly more similar but generally comparable for related versus unrelated mother-child pairs (Figures 2A and 3). This indicates that inherited maternal factors associated with the TCR recombination machinery are insufficient to yield the essentially similar TRBV gene segment selection in the child.

Remarkably, both within related and unrelated pairs, TRBV gene segment usage in low-frequency in-frame TCR beta clonotypes was more similar compared to that in the out-of-frame TCR beta variants (Figure 3). The equalization of the usage of TRBV gene segments in functional TCR variants (Figure 4) is probably a manifestation of selective pressure during thymic T cell selection, which should distinguish TRBV gene usage in functional TCRs from that preserved in unselected, out-of-frame TCR beta variants. This pressure on relative TRBV usage frequencies was prominent and led to significant convergence in both related (*P* = 0.0006) and unrelated (*P* = 0.0015) pairs, indicating that thymic selection essentially and similarly shapes the initial output of the TCR recombination machinery at the population level.

Interestingly, thymic selection also essentially filters out the longest CDR3 variants with large numbers of added nucleotides, as can be concluded from our comparison of non-functional out-of-frame and in-frame TCR beta CDR3 repertoires (Figure 7A).

Since TRBV gene segments encode the fragments of TCR chains that interact with MHC (33–35), we would expect that related mother-child pairs, being haploidentical (i.e., sharing at least 50% of HLA alleles), are characterized by more similar TRBV gene segment usage frequencies at the level of functional T cells compared to unrelated donor pairs due to the impact of identical HLA genes in thymic selection. Indeed, we observed that differences in TRBV gene segment usage in related versus unrelated pairs became more pronounced and statistically significant (*P* = 0.02) at the level of low-frequency, in-frame TCR beta CDR3 clonotypes (Figure 3). However, the general direction of TCR beta repertoire shaping was similar for related and unrelated donors, suggesting that the pressure of thymic selection is relatively homogenous in the population. The strength of this general pressure was far greater relative to the specific changes that were characteristic of related donors, which only added a minor codirectional trend (Figures 3 and 4).

The subset of high-frequency TCR beta clonotypes was characterized by increased variability in TRBV segment usage, and related and unrelated mother-child pairs were indistinguishable at this level (Figures 2C and 3). This is presumably due to the fact that different antigen specificities (but not TRBV segment interaction with MHC) play a dominant role in the priming and expansion of T cell clones, and this semi-random process negates the initial correlations that we observed in TRBV gene usage at the level of naïve T cells.

It should be noted, however, that the above analysis refers to low- and high-frequency clonotypes, which do not fully coincide with the naïve and antigen-experienced T cell subsets, respectively. It was previously demonstrated in other studies that recombinatorial biases might result in relatively high frequencies for certain naïve T cell clones, whereas some memory T cell clones may occur at relatively low frequencies (11, 14, 15). Moreover, these studies have shown a substantial overlap between the naïve and memory T cell repertoires, which suggests that a number of TCR beta CDR3 clonotypes could be associated with both subsets, being paired with either the same or alternative TCR alpha chains.

### Overlap of TCR beta repertoires

We observed the greatest relative overlap of TCR beta repertoires among high-frequency clonotypes. This observation can be explained by the presence of common expanded antigen-experienced clonotypes recognizing the same antigens, as well as of high-frequency naïve clonotypes carrying the TCR beta CDR3 sequence variants of low complexity that are repetitively produced in thymus and may be highly represented both within and between individuals (15).

In all comparisons, only slightly higher numbers of shared clonotypes were observed in related versus unrelated mother-child pairs (Figure 5). This observation is in agreement with the previous report by Robins et al. where the overlap in the naïve CD8+ CDR3 sequence repertoires was suggested to be independent of the degree of HLA matching based on results obtained from three related donors (14). Here, we have achieved a more accurate comparison by studying a larger cohort of related donors, using unbiased library preparation techniques, sequencing the samples being compared on separate Illumina lanes to protect from potential cross-sample contamination on the solid phase and performing deeper individual profiling. Even with these various methodological improvements, we still observed only a subtle trend toward increased TCR beta repertoire overlap in related individuals.

However, among the shared high-frequency amino acid CDR3 variants, the percentage of TRBV-CDR3 identical clonotypes was always higher for related pairs compared to unrelated ones, and correlated with the number of identical MHC-I alleles (Figure 6). This finding indicates that optimal recognition of the particular peptide-MHC complex often requires full functional convergence of the TCR beta chain, leading to an increased share of TRBV-identical common CDR3 variants in individuals carrying the same HLA alleles. Notably, this phenomenon was observed for bulk T cell populations, where the input of CD8+ T cells was sufficient to provide correlation. This correlation would probably be much higher if we were to specifically analyze sorted CD8+ T cells.

### Searching for persistent mature-microchimeric clones

In humans, maternal T cells are present in different fetal tissues (46, 48, 51), and may be present in the cord blood at a frequency of 0.1–0.5% of total T cells (48, 52). This can represent hundreds of thousands or millions of cells, of which many are likely to be memory T cells (52) capable of further clonal proliferation. Transmigration of maternal differentiated effector/memory Th1 and Th17 cells through the placenta was recently demonstrated in mouse models (53). Transfer of mature T cells is also possible in the opposite direction, and the presence of fetal microchimeric CD4+ and CD8+ T cells was registered in maternal blood during normal pregnancy in humans, predominantly in the third trimester (41) when mature α/β T cells are circulating in the fetus at significant numbers (54). Such mature-microchimeric T cell clones could further affect immunity to solid tumors (55, 56), influence transplantation tolerance (7), cause autoimmune diseases (3, 4, 43, 56–59), or protect the child against infections he/she has never encountered before.

Recent work has demonstrated that, in general, experienced clonal T cells commonly persist in the body for many years (17, 60). We have observed more than 20,000 TCR beta clonotypes that persisted in a patient for at least 7 years – from 2005 until 2012 – even after the patient underwent autologous HSC transplantation in 2009 [Ref. (16) and our unpublished data]. Similarly, naïve T cell clones persist in the body for many years after loss of thymus functionality (61). Therefore, if the engraftment of mature T cell clones transferred from mother to child and/or *vice versa* is a systemic process, we could expect to be able to verify the presence of such clones by using characteristic TCR beta CDR3 variants as clonal identifiers.

In our repertoire analysis, we did not observe mature-microchimeric T cell clones at a level of methodological sensitivity of ~100 mature-microchimeric clones per 10^6^ analyzed TCR beta clonotypes. Still, this does not preclude the existence of mature T cell-based maternal or fetal microchimerism at levels below the sensitivity achieved in the current study, in minor number of individuals, or in pathological conditions such as autoimmune disease.

It should be noted that deep TCR beta profiling methodology presently appears to be insufficiently sensitive for identifying particular expanded mature-microchimeric T cell clones, due to the general abundance of common identical TCR beta clonotypes. The following combination of methods could offer a potential way forward: (1) deep TCR beta profiling suggesting the presence of a particular expanded mature-microchimeric T cell clone, preferably with many added nucleotides within CDR3; (2) cell sorting using a TRBV family specific antibody in order to enrich for the hypothetical microchimeric clone of interest; and (3) real-time PCR confirmation of increased microchimerism in the sorted sample.

We also believe that further development of NGS profiling methods – especially in combination with the use of live cell-based emulsion PCR to identify paired TCR alpha-beta chains (62), and to potentially identify TCR beta chains paired with specific HLA molecules serving as an internal marker of microchimeric clones – should greatly facilitate future studies of mature T cell microchimerism in health and disease.

## Conflict of Interest Statement

The authors declare that the research was conducted in the absence of any commercial or financial relationships that could be construed as a potential conflict of interest.

## Footnotes

^1^http://mitcr.milaboratory.com/

^2^http://www.R-project.org/
